# Soemmering’s Ring in a Spontaneous Partially Absorbed Lens: A Case Report

**DOI:** 10.7759/cureus.25898

**Published:** 2022-06-13

**Authors:** Muhammad Syamil Bin Mohammad Salmi, Mazaya Binti Mahmud, Amirah Binti Razali, Rafidah Binti MD Salleh

**Affiliations:** 1 Department of Ophthalmology, Faculty of Medicine & Health Sciences, Universiti Putra Malaysia, Serdang, MYS

**Keywords:** ocular infection, aphakia, cataract extraction, cataract, absorbed lens, soemmering's ring

## Abstract

Soemmering's ring and spontaneous lens absorption are two distinct conditions that are uncommon and unlikely to occur simultaneously. We report a case of a 67-year-old man who presented with blurred vision in his left eye and has had poor eyesight since birth. His right and left eye visual acuities were 6/7.5 and hand movement (HM), respectively. There was no relative afferent pupillary defect (RAPD). The right eye’s examination was unremarkable. The left eye revealed a spontaneous rupture of the anterior lens capsule with partially absorbed lens material and the presence of Soemmering's ring. There was no evidence of phacodonesis. The left fundus appeared slightly hazy, while the retina appeared flat. Extraction of the left eye lens was performed for the patient, and he was left aphakic.

In this case, the patient's Soemmering's ring was linked to the ruptured anterior lens capsule followed by spontaneous partial absorption of lens material, which caused deposition of residual lens filaments near the equator of the capsule sac. In addition to ocular trauma, patients with congenital rubella infection of the eye, uveitis, and Morgagnian cataract have reported spontaneous absorption of lens material. The exact mechanism by which cataracts dissolve spontaneously is unlikely to be the same in all patients. This patient who has had an unsightly left eye since birth is presumed to have been born with an ocular infection complicated by amblyopia. The presence of both the Soemmering's ring and spontaneous lens absorption is unusual in this case. Early attention to the precious fellow eye is critical to ensure that the other unaffected eye maintains an adequate vision and allows independent patient mobility.

## Introduction

Literature reports that Soemmering's ring and spontaneous lens absorption are two distinct diseases [1,2] that are uncommon and unlikely to occur concurrently in a single patient. Soemmering's ring was first discovered in 1928 by Soemmering as the deposit of residual equatorial lens' epithelial cells. These residual cells continue to proliferate and create new cortical fibers that eventually form a ring between the posterior capsule and the borders of the anterior capsule remnants [3].

J.C. Saunders first identified spontaneous lens absorption in 1811. He believed the condition was connected with a congenital cataract. Subsequent case reports that agreed with Saunder’s findings further described that each case had a unique mechanism. Lens absorption can either occur independently or be associated with maternal rubella, leptospirosis, uveitis, persistent hyperplastic primary vitreous (PHPV), Hallerman-Streif-Francois syndrome, Down syndrome, trauma, and a variety of other factors [2]. We report a rare case of Soemmering’s ring in a spontaneous partially absorbed lens, with a description of a surgical-based treatment.

## Case presentation

The case-patient was a 67-year-old gentleman who presented with a blurring of vision of his left eye without any pain. The patient has had poor eyesight since birth, denied of any past trauma or ocular surgery on the left eye, and suffers from diabetes mellitus complicated with nephropathy and dyslipidemia.

**Figure 1:**
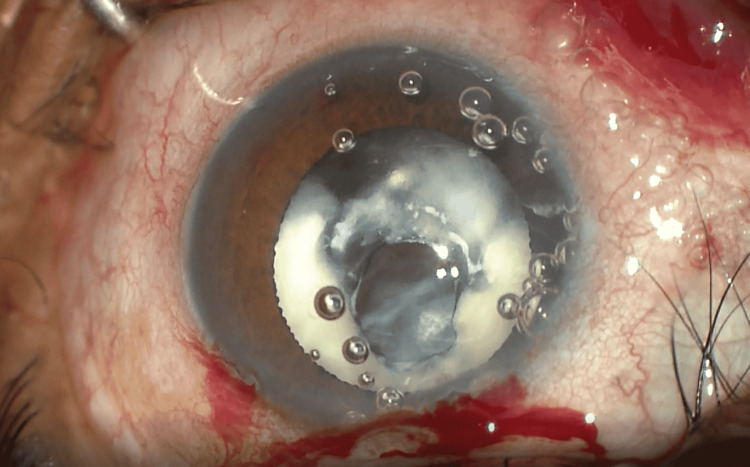
Primary rupture of the anterior lens capsule with surrounding Soemmering's ring

Visual acuity for his right and left eyes was 6/7.5 and hand movement (HM), respectively. Upon examination of the right eye, relative afferent pupillary defect (RAPD) was not present, and the anterior segment was normal. However, there was a spontaneous rupture of the anterior lens capsule, with a partially absorbed lens material, and the presence of Soemmering’s ring in his left eye without phacodonesis (Figure 1). No old keratic precipitates or posterior synechia were present. Intraocular pressure was 16 mmHg in the right eye and 23 mmHg in the left eye.

On dilated fundus examination, the right eye was unremarkable with a cup-to-disk ratio of 0.3. The right eye also had normal macula and retina appearances, without diabetic-related changes. The left fundus, however, appeared slightly hazy, while the retina appeared grossly flat.

The patient was planned for extraction of the left eye lens and the Soemmering’s ring. He was informed that the eye could be left aphakic from the procedure and may require a secondary intraocular lens (IOL) implantation depending on his visual potential during a post-operative assessment.

The patient was prepared for a routine lens extraction under local anesthesia. A paracentesis was made at 2 o’clock, and the main wound was created at the superior cornea. An ophthalmic viscosurgical device (OVD) was deliberately injected through the anterior capsule opening to gently inflate the bag and better assess the actual condition of the lens.

**Figure 2:**
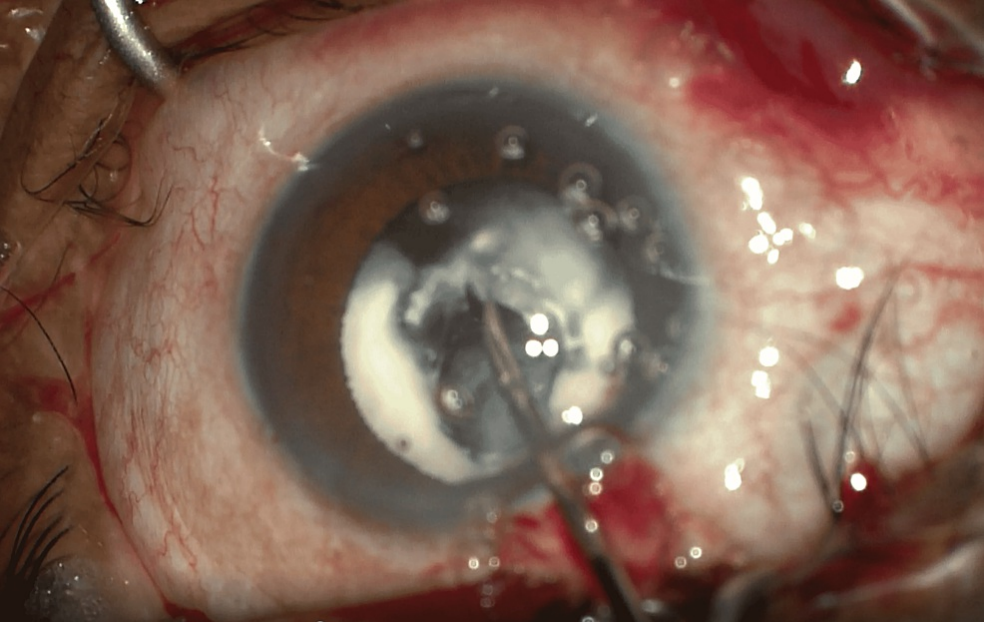
Refashioning of primary anterior lens capsule rupture using microscissors

After successful inflation of the bag, zonulodialysis was noticed from 2 to 6 o’clock, along with a calcified posterior capsule. The torn anterior capsule was further refashioned to resemble an adequately sized continuous curvilinear capsulorhexis (Figure 2). Primary rupture of the posterior capsule was also noticed. Attempts were made to remove the residual lens material with a manual Simcoe irrigation/aspiration cannula, followed by the usage of a vitrectomy cutter.

**Figure 3:**
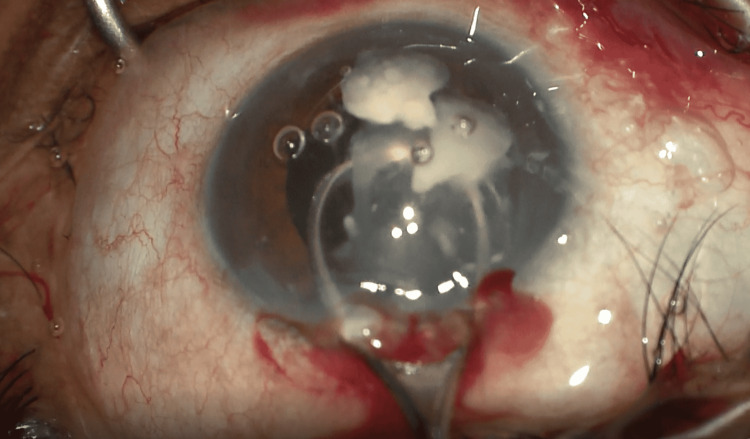
Manual removal of fragments from the Soemmering's ring using a Vectis

**Figure 4:**
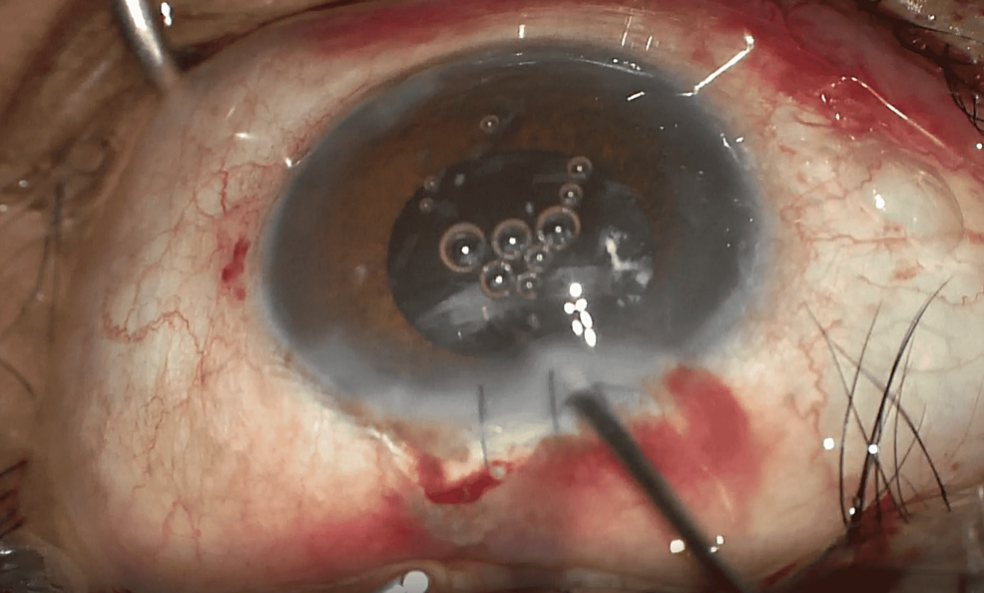
Surgeon attempting to remove calcified superior cortical matter using a vitrectomy cutter

Next, the rigid part of the Soemmering’s ring was manually manipulated and removed using the bimanual technique. During the procedure, a few segments of the ring were too dense and could not be cut and aspirated using the vitrectomy cutter. Therefore, these segments were made to float into the anterior chamber using the OVD and removed with Vectis through an enlarged cornea wound (Figure 3). The dense but softer lens materials were carefully removed with a Simcoe cannula. To prevent fragments from getting into the vitreous cavity through the posterior capsule opening, the OVD was regularly used to block the rent. Additionally, the peripheral cortical material attached to the capsule was removed, and refashioning of the posterior capsule rent was attempted. However, the superior segment had dense calcification and could not be removed after multiple attempts (Figure 4). An IOL was not implanted during the surgery.

The patient’s left eye vision remained at HM three days post-surgery. The left fundus appeared normal, with a pink optic disk, a cup-to-disk ratio of 0.3, and a healthy retina. A topical steroid and an antibiotic were prescribed according to the routine post-cataract surgery regime. A refractive assessment one month after surgery showed that the best-corrected visual acuity of the left eye persisted at HM, which was due to dense amblyopia. The patient was scheduled for a neodymium-doped yttrium aluminum garnet (Nd:YAG) laser capsulotomy at a later follow-up period.

## Discussion

A Soemmering's ring is commonly made up of several kinds of organic matter, including lens fiber material, recrystallized lens protein, amorphous matter, and cellular material, particularly at the subcapsular layer. Due to these characteristics, the condition is also known as a secondary cataract formation [4]. The true appearance of the ring is prominently seen during its intraoperative extraction.

The formation of this ring usually occurs after cataract surgery with IOL implantation when the central region of the anterior lens capsule is exposed. This results in the deposition of lens filaments near the equator of the capsule sac that is not absorbed [5]. The Soemmering's ring of the patient in this case report was linked to the spontaneous absorption of lens material with rupture of the anterior lens capsule, which is a rare occurrence.

In addition to ocular trauma, spontaneous absorption of lens material has been reported in patients with congenital rubella infection of the eye, uveitis, and Morgagnian cataract. However, the exact mechanism by which cataracts spontaneously dissolve is unlikely to be identical in all patients [6-8]. The patient in this study is believed to be born with an ocular infection complicated with amblyopia, and as a result of the illness, he had been left with an unsightly left eye since.

The surgical technique used to remove the Soemmering’s ring in this case report was adapted from a method described by Gimbel et al. with slight modifications. The original procedure was successful in implanting an IOL in the OVD bag, following the removal of the lens material [9]. Implanting an IOL in this patient may not have been the best option as the patient was likely to have developed amblyopia since early childhood.

## Conclusions

The simultaneous presence of both the Soemmering's ring and spontaneous lens absorption in this case report is unique and rare. To the best of our knowledge, this simultaneous occurrence is rarely reported previously. It is postulated that the patient’s neglected congenital ocular infection that had progressed to amblyopia caused the Soemmering's ring and spontaneous lens absorption to occur concurrently. Early attention to the precious fellow eye is crucial to ensure that the other unaffected eye maintains an adequate vision and allows independent patient mobility.
